# Holding the Inflammatory System in Check: TLRs and Their Targeted Therapy in Asthma

**DOI:** 10.1155/2016/2180417

**Published:** 2016-05-04

**Authors:** Zhiyong Dong, Lingxin Xiong, Weijie Zhang, Peter G. Gibson, Ting Wang, Yanjiao Lu, Guoqiang Wang, Hui Li, Fang Wang

**Affiliations:** ^1^Department of Pathogen Biology, Basic Medical College, Jilin University, Changchun 130021, China; ^2^Department of Respiratory Disease, Jilin Provincial People's Hospital, Changchun 130021, China; ^3^Department of Respiratory and Sleep Medicine, John Hunter Hospital, Newcastle, NSW 2305, Australia

## Abstract

Inflammation is a complex biological response to detrimental stimuli and can be a double-edged sword. Inflammation plays a protective role in removing pathogenic factors, but dysregulated inflammation is associated with several major fatal diseases such as asthma, cancer, and cardiovascular diseases. Asthma is a complex heterogenous disease caused by genetic and environmental factors. TLRs are the primary proteins associated with the innate and adaptive immune responses to these fatal factors and play an important role in recognizing pathogen-associated molecular patterns (PAMPs) and damage-associated molecular patterns (DAMPs), which initiates the downstream immune response. Due to the complex TLRs cascade and nowadays unsuccessful control in asthma, new studies are focused on TLRs and other potential targets in TLR cascade to minimize airway inflammation.

## 1. Introduction

Inflammation is a complex host response to detrimental stimuli including tissue injury, microbial infection, and irritant exposure. It is classically characterized by redness, swelling, heat, pain, and tissue dysfunction [1]. When inflammation involves mucosal surfaces, there are accompanying mucus hypersecretions and epithelial shedding. Inflammation plays a protective role in the body in negating pathogenic factors such as microbial infections and oxidative stress and is a healing process enabling repair of damaged tissue [2]. On the contrary, persistence of inflammation with overproduction of cytokines by immune cells including macrophages, neutrophils, eosinophils, dendritic cells, mast cells, natural killer cells, and structural cells such as endothelial cells, mucosal epithelial cells, and fibroblasts can be harmful. Dysregulated inflammation is associated with several diseases including asthma, cancer, cardiovascular disease, autoimmune diseases, and metabolic disease.

Asthma is a complex heterogeneous disease associated with local tissue chronic inflammation of the airway and is characterized by variable and recurring symptoms (including wheezing, coughing, chest tightness, and shortness of breath), reversible airflow obstruction, airway remodeling, and airway hyperresponsiveness. According to Chung [3], asthma is ranked as the 14th most important chronic disease worldwide regarding the prevalence, extent, and duration of disability and affects 334 million individuals of all ages, resulting in 90 and 170 deaths per million in female and male individuals, respectively. In addition, asthma causes a heavy economic burden for the government and individuals. For example, in Europe, total cost per patient ranges from €509 for controlled asthma to €2281 for uncontrolled asthma [4]. Asthma is caused by a complex and incompletely understood combination of genetic (polymorphisms of multiple genes) and environmental (such as respiratory infections and particulates PM2.5) factors, which induce an immune response via the infiltration of inflammatory cells into the airway and consequent cytokine release. Emerging evidence shows that Toll-like receptors (TLRs) are associated with the inflammatory response and chronic airway inflammation in asthma [5]. TLRs are a subgroup of pattern recognition receptors (PRRs) that are expressed by cells of the innate immune system and that sense two classes of molecules such as pathogen-associated molecular patterns (PAMPs) and damage-associated molecular patterns (DAMPs), which then initiates the downstream immune cascades.

Many previous studies have focused on the discovery, structure, and roles of TLR family members and related signaling pathways in airway diseases, but few studies emphasize TLR expression in asthma, especially in the different phenotypes. This review will highlight the roles of TLR members in airway inflammation and their association with the pathogenesis of distinct asthma phenotypes and in addition will discuss the potential for TLR-targeted therapies in the treatment of asthma.

## 2. TLRs Family and Related Signal Pathways

Toll-like receptors (TLRs) are a class of single, transmembrane, and noncatalytic proteins named PRR and are expressed on specific immune cells (i.e., macrophages and dendritic cells) as well as nonimmune cells (e.g., epithelial, fibroblast, and endothelial cells) [6]. TLRs bind to and recognize endogenous molecules named DAMPs (e.g., structurally conserved components of microbes) and exogenous molecules that are named PAMPs (e.g., viral and bacterial products). Additionally, after the recognition by TLRs, downstream cascades are initiated. TLRs are involved in the initiation of innate immune responses and play a protective role against microbial infections. Once microbes invade physical barriers such as the skin or intestinal tract mucosa, TLRs on the cellular surface respond to microbial membrane materials (e.g., lipids and lipoproteins) and intracellular TLRs recognize microbial nucleic acids to initiate a host response [7].

So far, a total of 10 TLR genes in humans (TLR1–TLR10) and 12 (TLR1–TLR9 and TLR11–TLR13) in mice have been discovered. The 10 TLRs family members in humans are categorized into two subgroups. The first subgroup that recognizes the components of microbial membranes includes TLR1, TLR2, TLR4, TLR5, TLR6, and TLR10 of humans and TLR11 and TLR12 of mice and is primarily expressed on the cell surface [8]. The second subgroup that responds to microbial nucleic acids includes TLR3, TLR7, TLR8, and TLR9 and is expressed intracellularly in vesicles (e.g., lysosomes, endosomes, and the endoplasmic reticulum). TLR signaling is divided into two distinct signaling pathways, that is, the myeloid differentiation factor 88- (MyD88-) dependent and Toll/IL-1 receptor-domain containing adapter-inducing interferon-*β*- (TRIF-) dependent pathway. Both pathways are involved in innate immunity. MyD88 and TRIF bind independently to TLRs, resulting in the production of cytokines such as TNF-*α*, IL-1*β*, IL-6, and type I IFNs [9].

### 2.1. MyD88 Pathway

MyD88 possesses an amino- (N-) terminal death domain (DD), a shorter linker sequence, and a carboxy- (C-) terminal Toll/interleukin-1 receptor (TIR) domain. MyD88 also has an intermediate domain (ID) that interacts with IL-1R-associated kinases 4 (IRAK4) in TLR signaling [10, 11]. MyD88-dependent signaling is used by all TLRs except TLR3. The knockout of MyD88 in mice showed no responses to the ligands of TLR family members including TLR2, TLR4, TLR5, TLR7, and TLR9, indicative of the key role of MyD88 in TLRs-mediated inflammatory responses [12–16].

### 2.2. TRIF Pathway

TRIF is a large protein containing 712 amino acids in humans and directly binds to TLR3 and indirectly binds to TLR4 via connection with another adaptor protein, TRIF-related adaptor molecule (TRAM) [10]. The knockout of TRIF in mice triggers defective expression of IFN-*β* production and IFN-related genes that are mediated by TLR3 and TLR4, although early-phase activation of NF-*κ*B and TLR4-mediated activation of MyD88 pathway were observed [17]. Similarly, TRIF was confirmed to have a key role in the induction of inflammatory mediators contributing to antiviral innate immune responses via MyD88-independent signaling that is mediated by both TLR3 and TLR4 [18].

## 3. What Is Asthma?

Asthma is a common heterogeneous disease characterized by chronic airway inflammation and is defined by recurring respiratory symptoms (such as wheezing, cough, shortness of breath, and chest tightness) that vary over time and in intensity, as well as by airflow obstruction according to GINA report [19]. Asthma causes a serious global health threat to patients of all age groups and is increasing in many countries in its prevalence, especially among children. Some countries have experienced a significant decline in hospitalizations and mortality from asthma; however, asthma still imposes a heavy burden on public health systems and on society through productivity decreases.

Due to both exposures (such as allergen and microbial infection) and treatment, there is heterogeneity in the inflammatory response in asthmatic airway. Wang et al. [20] previously categorized asthma into four phenotypes such as neutrophilic, eosinophilic, mixed granulocytic, and paucigranulocytic asthma according to inflammatory cell counts in induced sputum. Individualized precise diagnosis and treatment based on inflammatory phenotypes are now advocated because of limitations on the premise of current management of asthma. Individualized therapy is the customization of health care tailored to the individual and uses previously infeasible technologies based primarily on recent cluster analyses, molecular phenotyping, biomarkers, and differential responses to therapies, distinguishing a given patient from other patients with similar clinical presentations [21, 22]. Nowadays, the mainstay of asthma treatment is daily long acting *β*_2_ agonists and inhaled corticosteroids (LABA/ICS) [3]. Maintenance treatment with LABA/ICS relieves asthma symptoms and reduces the frequency of exacerbations; however, there are limits in treatment options for people who do not gain control on combination LABA/ICS [23]. Targeted therapies at IgE, interleukin-4 (IL-4), IL-4 receptor, IL-5, IL-13, tumor necrosis factor-*α*, and CRTh2 are new treatment paradigms for asthma [24]. Emerging studies demonstrate that TLRs-targeted therapies potentially play a key role in effectively controlling airway inflammation in asthma.

## 4. TLRs in Asthma

### 4.1. TLR2 and TLR4 in Neutrophilic Asthma

**Figure 1:**
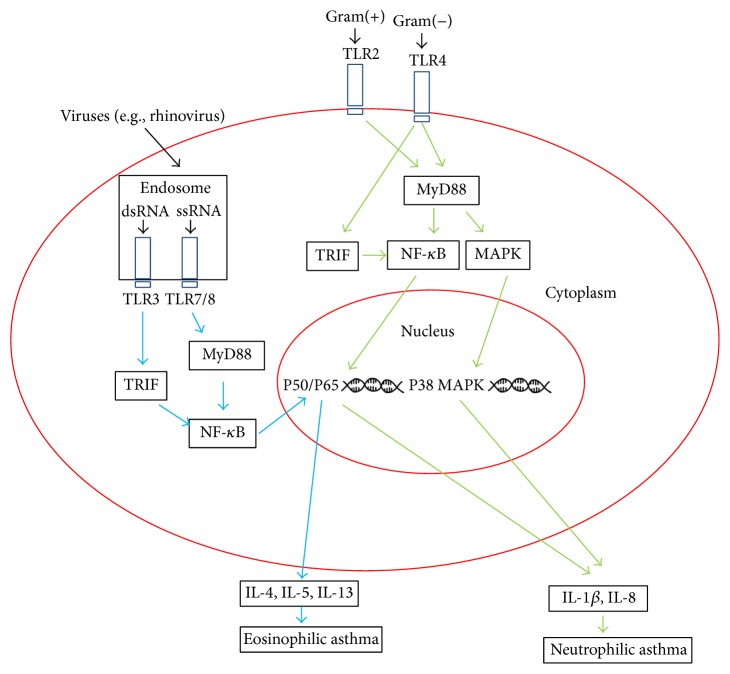
Schematic overview of TLR signaling pathway in neutrophilic and eosinophilic asthma. Gram-negative and Gram-positive bacteria as well as respiratory viruses (e.g., rhinovirus) are detected by TLRs. Subsequently, TLR3 and TLR7/8 trigger TRIF and MyD88 pathways, respectively, followed by the transcription of NF-*κ*B in nucleus and the production of IL-4, IL-5, and IL-13, inducing eosinophilic asthma. TLR2 and TLR4 induce MyD88 and MyD88 as well as TRIF cascades, respectively, followed by the transcriptions of MAPK and NF-*κ*B into nucleus, triggering the release of IL-1*β* and IL-8 and the onset of neutrophilic asthma. MAPK: mitogen-activated protein kinase; MyD88: myeloid differentiation primary-response gene 88; NF-*κ*B: nuclear factor-*κ*B; TLR: Toll-like receptor; TRIF: Toll/IL-1R (TIR) domain containing adaptor protein inducing IFN-*β*.

The role of adaptive immune responses in asthma is well studied and involves T helper type 2 lymphocyte activation by allergen, accompanied by eosinophilic airway inflammation. The innate immune system is also associated with the pathogenesis of asthma and the onset of inflammation in the airway. Simpson first discovered that an upregulation of the innate immune receptors TLR2 and TLR4 as well as proinflammatory cytokines IL-8 and IL-1*β* was involved in neutrophilic asthma [25]. TLR2 plays an important role in recognizing Gram-positive bacteria and TLR4 is responsible for the detection of Gram-negative bacteria through their microbial components such as lipopolysaccharides (LPS) (Figure 1) [8].

### 4.2. TLR7 and Eosinophilic Asthma

TLR7 is intracellularly expressed on the surface of airway epithelia and airway smooth muscle as well as innate immune cells (such as macrophages, natural killer cells, and dendritic cells) [26, 27] and plays a significant role in the pathogenesis of autoimmune disorders such as Systemic Lupus Erythematosus (SLE) and in the regulation of antiviral immune responses [28]. TLR7 recognizes single-stranded RNA, a common molecular component to respiratory viruses, resulting in regulating downstream interferon production and the activation of Th1 antiviral responses [28]. TLR7 exhibits its antiviral activity in combination with TLR8, the homologue of TLR7 that also recognizes single-stranded viral RNA (Figure 1).

TLR7 plays an important role in reduction of airway inflammation, promoting Th1 responses in immune cells, reversing airway hyperresponsiveness, and preventing airway remodeling. Airway inflammation is essential to the pathogenesis of asthma and is triggered by respiratory viral infections and inhaled allergen, leading to the activation of T helper 2 (Th2) cell differentiation and the secretion of Th2 cytokines such as IL-4, IL-5, and IL-13 [29]. IL-5 matures eosinophils in the bone marrow and, together with chemokines such as eotaxins, promotes recruitment of eosinophils into the airways, resulting in local eosinophilic inflammation [30]. TLR7 stimulation suppresses eosinophilic airway inflammation in a variety of animal models of asthma through reducing Th2 cytokines such as IL-4 and IL-5 as well as eotaxin in the lung [31] and IgE [32]. On the other hand, IL-5 induced airway eosinophilia can act as negative regulator of TLR7 expression and antiviral responses [30]. The role of TLR7 is not limited to Th2 responses; besides, it is involved in Th1 responses in immune cells. TLR7 activation promotes the reduction of Th2 cells and the enhancement of Th1 cells, which results in increases in Th1-cytokine release and decrease in IgE production [33–35], exhibiting the immunomodulatory activity of TLR7 in maintaining Th2/Th1 balance. IL-13 is responsible for inducing airway hyperreactivity (AHR) and mucus production in eosinophilic asthma [36]. TLR7 stimulation ameliorates ovalbumin-induced AHR when animals are treated with TLR7 agonists. A number of emerging studies suggest that the suppression of AHR involves NF-*κ*B and p38 MAP intracellular signaling and is dependent on iNKT cells and IFN-*γ* production [26, 37]; however,* in vivo* investigation on the mechanism of AHR amelioration remains incomplete. Additionally, TLR7 ligand prevents chronic irreversible asthmatic airway remodeling including smooth muscle proliferation and goblet cell hyperplasia [38, 39].

### 4.3. TLR Genetic Polymorphisms and Asthma

Genetic polymorphisms in TLRs may be responsible for individual susceptibility and severity of asthma. Genetic diversity in specific alleles determines the differences in susceptibility to a specific disease to some extent [40]. Polymorphisms in the TLR4 gene affect sensitivity to allergens [41, 42]. Zhang et al. [42] discovered a harmful effect of the TT homozygote allele in the TLR4 gene rs1927914 on the forced expiratory volume in 1s (FEV_1_), implicating impaired lung function. Additionally, the AA homozygote genotype and A allele in Asp299 Gly of the TLR4 gene may correlate with a reduced asthma risk, as indicated by the association between TLR4 polymorphisms and the development of asthma in the study by Tizaoui et al. [43]. In addition to TLR4, variants of the TLR2 gene were reported to have some association with childhood asthma in Caucasians [44], and TLR2 polymorphism affects the asthma risk and lung function [45]. It has been shown that variants in the TLR7/8 genes as well as the TLR10 gene showed no significant association in some alleles despite the relevance between other polymorphisms in the TLR10 gene and asthma [42, 45–48]. In terms of TLR1 and TLR5, studies on the association between genetic polymorphisms and the development of asthma have not been reported. Future investigations should emphasize TLR genetic variants such as haplotype analysis and gene-environmental interaction [43].

### 4.4. TLR and Viral Infection

Viral infection is a common acute trigger of asthma and exacerbation of asthma. Approximately 80% of asthma exacerbations are caused by respiratory viral infection [49, 50]. The PRRs in the detection of viral infection include TLR7 and TLR8 which detect single-stranded RNA and TLR3, retinoic acid-inducible gene I (RIG I), and melanoma differentiation associated gene 5 (MDA5) that are activated by double-stranded RNA. TLR7 expression is associated with the severity of the disease [51]. Airway cells from asthmatic patients are vulnerable to viral infection due to impaired innate antiviral responses compared to healthy subjects. This vulnerability is triggered by aberrant production of type I IFN, an antiviral cytokine [51]. TLR7 deficiency was discovered in alveolar macrophages from severe asthmatic and affected the interferon responses to rhinovirus infection. In the same study, the abnormal expression of the three microRNAs such as miR-150, miR-152, and miR-375 was the trigger of TLR7 deficiency. When these miRs were blocked, this resulted in restored TLR7 expression and augmented interferon responses to rhinovirus infection, indicating that TLR7 is associated with the vulnerability of asthmatic subjects [51]. In addition to this finding,* in vivo* research shows that a lack of TLR7 signaling in a rhinovirus-induced asthma exacerbation leads to reduced IFN production and exaggerated Th2-driven inflammation, suggesting the role of TLR7 signaling in rhinovirus-induced asthma exacerbation [30]. Other investigations support this finding. Bronchoalveolar lavage (BAL) cells from nonsevere asthma possess a deficient IFN response to rhinovirus infection [52, 53]; additionally, TLR7 dysfunction was shown in asthmatic peripheral blood mononuclear cells [54]. TLR3 also detects double-stranded RNA genome of respiratory virus which represents the replication of RNA viruses and protects the host by the induction of inflammatory responses including type I IFN production and activation of NK cells and cytotoxic T lymphocytes [55]. In an investigation by Parsons et al. [56], although no difference in the expression of TLR3 was observed, primary bronchial epithelial cells (pBECs) from asthmatics demonstrated an ineffective innate immune response following RV infection, with impaired type I and type III interferon responses to the infection. In addition, RV infection of healthy pBECs triggered a robust upregulation of TLR3, while inhibition of TLR3 signaling leads to a marked inhibition of both type I and type III interferon responses.

### 4.5. TLR9 and Asthma

TLR9 is intracellularly expressed in the immune cells such as B lymphocytes, monocytes, and plasmacytoid dendritic cells and detects unmethylated CpG motifs in microbial DNA molecules [57]. In allergic asthma subjects, TLR9 expression on plasmacytoid dendritic cells and TLR9-induced responses are upregulated by IL-25 that originates from airway epithelial cells [58]. In an* in vivo* investigation in severe asthma, Duechs et al. [59] discovered that TLR9 activation significantly reduced some features of the asthmatic phenotype such as a reduction in eosinophil influx and IgE levels in serum. The same study also observed a decreased release of cytokines such as IL-4, IL-5, IFN-*γ*, IL-1*β*, and IL-12, indicative of enhanced Th1 response, suggesting that TLR9 activation may suppress the Th2 response via promoting a Th1 response. Similarly, a Th1 response induced by the exposure to CpG DNA opposes a Th2 response in a murine model of asthma [60]. TLR9 is also involved in the inhibition of airway remodeling [61–64], suggesting a potential protective role of TLR9 in asthma. This was evaluated in* in vivo* models where TLR9 activation was found to be associated with a reduction in antigen-induced respiratory allergic responses [65, 66], suggesting that TLR9 ligands could be used as prophylactic and therapeutic agents in asthma [67]. However, TLR9 targeted treatment was not found to be efficacious in preexisting severe allergic inflammation in the airway, in either animal experiments or clinical trials [68, 69]. The role of TLR9 agonists in asthma requires further evaluation.

## 5. TLRs Targeted Therapeutics

### 5.1. Effect of TLR Agonists in Asthma

The typical treatment for asthma and asthma exacerbations includes inhaled corticosteroids for their ability of enhancing *β*-adrenergic responses and repressing inflammation in airways [70]. Nevertheless, in the treatment of severe asthma, corticosteroids are ineffective in alleviating symptoms, probably because oxidative stress as well as subsequent DNA damage leads to decreased activity of transcriptional corepressors such as histone deacetylase-2 (HDAC-2) [8]. Recently, TLRs agonists have been considered as agents in controlling asthma. TLRs agonists can be categorized into cellular surface TLRs agonists and intracellular TLRs agonists based on the distribution of TLRs. Cell surface TLRs sense structural components of microbia ranging from Gram-positive bacteria to Gram-negative bacteria and some respiratory viruses in the onset and development of asthma and asthma exacerbation [67]. Targeting TLR4 to treat asthma is based on the activation of TLR4 as an adjuvant in allergy vaccines to induce tolerance and inhibition of TLR4 expression. TLR4 agonists such as MPL® (monophosphoryl lipid A) seem to work effectively as allergy vaccines due to overexpression of TLR4 in asthmatic patients [8]. Another cell surface TLRs agonist is Pam3CSK4 that acts as a synthetic TLR2 agonist and exhibits antiasthmatic effects by reducing Th2 cytokine release, AHR, IgE levels, airway inflammation, and asthmatic symptoms in animal models of asthma [67]. Intracellular TLRs agonists such as TLR7/TLR8 agonists have also been evaluated in asthma. Resiquimod is a typical TLR7/TLR8 agonist and* in vivo* suppresses AHR as well as airway remodeling in asthma [31, 39, 71–73]. In addition, this drug was also found to suppress both Th1 and Th2 cytokine production in the lungs of experimental animals and decrease lung eosinophilia, goblet cell hyperplasia, and IgE levels [39, 67, 71]. Many other agents that target TLRs have been found to control airway inflammation, eosinophilia, and AHR in distinct animal models of allergic inflammatory diseases [67]. It is obvious that in the future a wide variety of TLR agonists are likely to be evaluated as effective asthma controllers. On the contrary, future emphasis should be on the side effects of TLR agonists, especially on asthmatic children due to a lack of investigation on allergic children. Nowadays clinical trials are mainly conducted in adults, and besides uncertain targeting of the immature immunity in children as well as timing, dosage, and patient selection regarding the formulation to best employ TLRs agonists still needs further studies, which may hinder wider application of TLRs agonists.

### 5.2. Effect of Corticosteroid on TLR Expression

Corticosteroids are the most effective agents in inflammation management in asthma, and classical corticosteroids such as budesonide are recommended by guidelines for asthma treatment [74]. When inhaled corticosteroids (ICS) were introduced into asthma management, symptom control of asthma and lung function were improved, and exacerbations and asthma-related mortality decreased [19]. Corticosteroids influence TLRs and can upregulate TLR4 expression* in vivo* in peripheral blood mononuclear cells from asthmatic patients [75]. In addition to this finding, after* in vitro* stimulation with LPS, the production of both TNF-*α* and IFN-*γ* in PBMC supernatant was significantly increased by oral corticosteroids [75]. Similarly, Pace et al. [76] reported that TLR4 and TLR2 expression were increased in Treg lymphocytes from allergic asthmatic subjects after budesonide treatment compared to healthy controls, providing further understanding of the action mechanism of budesonide on the control of inflammation in asthma. Furthermore, an increased level of IL-10 and decreased level of IL-6 and TNF-*α* were observed after budesonide administration, confirming the modulatory potential of budesonide in immune responses to allergic subjects.

## 6. Conclusions

The invasion of antigens into airways causes the activation of PRRs such as TLRs in response to PAMPs. TLRs play an important role in the detection of invading pathogens by the innate immune system, and a total of 10 TLRs family members have been discovered in humans (TLR1–TLR10). TLRs induce the activation of MyD88 and TRIF signaling pathways, resulting in the production of inflammatory mediators via the NF-*κ*B pathway. Different pathogens trigger distinct immune activation of TLRs. TLR2 plays an important role in recognizing Gram-positive bacteria and TLR4 is responsible for the detection of Gram-negative bacteria, leading to the production of cytokines such as IL-1*β* and IL-8 and to the infiltration of neutrophils in asthmatic airways. In addition, TLR7 senses single-stranded viral RNA which inhibits Th2 immune responses and eosinophilic asthma, and TLR9 detects unmethylated CpG motifs in microbial DNA molecules in the development of asthma and asthma exacerbation. Furthermore, genetic polymorphisms affect the susceptibility and severity of asthma, making effective control of airway inflammation in asthma more complex. Nowadays, corticosteroid therapy is commonly used for asthma treatment, and some findings confirmed the modulatory role of corticosteroid in the mediation of TLR expression in asthmatic subjects. Combination therapy of corticosteroid and TLRs agonists may be potentially more effective in controlling inflammation in asthmatics compared to the traditional treatment by corticosteroid. However, the timing, dosage, patient selection, and many other questions regarding the formulation to best employ TLRs agonists remain unclear, and future work needs to address these difficulties in order to hold airway inflammation in check in asthma.
